# Understanding disorder in monolayer graphene devices with gate-defined superlattices

**DOI:** 10.1088/1361-6528/ad7853

**Published:** 2024-09-18

**Authors:** Vinay Kammarchedu, Derrick Butler, Asmaul Smitha Rashid, Aida Ebrahimi, Morteza Kayyalha

**Affiliations:** 1 Department of Electrical Engineering, The Pennsylvania State University, University Park, PA 16802, United States of America; 2 Materials Research Institute, The Pennsylvania State University, University Park, PA 16802, United States of America; 3 Department of Biomedical Engineering, The Pennsylvania State University, University Park, PA 16802, United States of America

**Keywords:** superlattice, graphene, disorder

## Abstract

Engineering superlattices (SLs)—which are spatially periodic potential landscapes for electrons—is an emerging approach for the realization of exotic properties, including superconductivity and correlated insulators, in two-dimensional materials. While moiré SL engineering has been a popular approach, nanopatterning is an attractive alternative offering control over the pattern and wavelength of the SL. However, the disorder arising in the system due to imperfect nanopatterning is seldom studied. Here, by creating a square lattice of nanoholes in the SiO_2_ dielectric layer using nanolithography, we study the SL potential and the disorder formed in hBN-graphene-hBN heterostructures. Specifically, we observe that while electrical transport shows distinct SL satellite peaks, the disorder of the device is significantly higher than graphene devices without any SL. We use finite-element simulations combined with a resistor network model to calculate the effects of this disorder on the transport properties of graphene. We consider three types of disorder: nanohole size variations, adjacent nanohole mergers, and nanohole vacancies. Comparing our experimental results with the model, we find that the disorder primarily originates from nanohole size variations rather than nanohole mergers in square SLs. We further confirm the validity of our model by comparing the results with quantum transport simulations. Our findings highlight the applicability of our simple framework to predict and engineer disorder in patterned SLs, specifically correlating variations in the resultant SL patterns to the observed disorder. Our combined experimental and theoretical results could serve as a valuable guide for optimizing nanofabrication processes to engineer disorder in nanopatterned SLs.

## 1. Introduction

The ability to engineer the potential landscape for electrons in two-dimensional (2D) materials is an emerging strategy to study exotic phases of matter [1], ranging from unconventional superconductors [2] and correlated insulators [2] to Wigner crystals [3] and Chern insulators [4–7]. The realization of these devices is possible due to the formation of a periodic potential by superlattice (SL) engineering. Several methods have been explored for SL engineering in 2D materials, including periodic heteroatom doping [8], pattern etching [9], moiré pattern (twist angle engineering or lattice mismatch) [10–18], strain engineering [19], ferroelectric domain gating [20], and patterned electrostatic gating [9, 21–29]. Although moiré heterostructures have been widely popular, they face several reliability problems. One of the main issues is the disorder originated from inhomogeneous angle and strain [29]. Further complications due to domain formation and lattice relaxation also affect the reproducibility of these devices [30]. On the other hand, patterned electrostatic gating allows for user-defined geometry (e.g. triangular, square, etc) and variable SL sizes. Furthermore, the SL formed using a periodic electrostatic field allows for the *in-situ* control and modulation of the SL potential [31].

SL engineering of hBN-encapsulated graphene devices via periodic electrostatic fields has been achieved by patterning a dielectric [23] between graphene and the gate [25], as well as using a patterned gate electrode with a uniform dielectric material [9, 32]. In both cases, the periodic electric field can have a controlled SL pattern and wavelength. Furthermore, the strength of the SL potential can be tuned on demand [25]. However, SL engineering via patterning is mostly limited by the nanofabrication process [9]. One of the challenges in fabricating patterned SLs via nanolithography is the control of SL pattern disorder which often limits the visibility of the SL effects during measurements [33]. In experiments, state-of-the-art encapsulated graphene devices usually have a residual carrier concentration ($n_0^{\prime}$) in the range of ∼10^10^ cm^−2^ [34]. This sets the disorder energy scale at about $\hbar {v_{\text{F}}}\sqrt {\pi n_0^{\prime}} $ ∼ 10 meV, where $\hbar $ and ${v_{\text{F}}}$ are the reduced Planck constant and the Fermi velocity, respectively [33]. However, due to variations in device fabrication processes, residual concentrations on the order of 10^11^ to 2.5 × 10^11^ cm^−2^ are common [35, 36], resulting in a disorder energy as large as 50 meV [33]. The SL effects have been predicted to emerge at such a large disorder level, albeit for nanopatterns with dimensions of a few tens of nanometers [29]. This size limitation is approaching the limit of what is possible using nanofabrication methods such as electron beam lithography (EBL) [9]. Furthermore, lithography itself could introduce variations in the device structure and add to the disorder of the system [33, 36–38]. It is, therefore, imperative to study and understand disorder effects in nanofabricated SLs to curate highly efficient fabrication processes. Previous studies have considered the variations produced by lithography in device structures that directly etch the graphene channel [39–42]. However, the impact of disorder is yet to be investigated in graphene heterostructures with nanopatterned dielectric substrates.

In this work, we study SL effects and disorder in hBN-encapsulated monolayer graphene [35, 43] devices with a graphite top gate and a patterned SiO_2_ dielectric layer. To investigate the SL effects, we perform low-temperature electrical transport and Hall measurements and observe that by increasing the SL potential, satellite SL peaks appear in the resistance versus carrier concentration data. In addition, our data show that the disorder in the patterned SiO_2_ graphene is an order of magnitude higher than that in unpatterned SiO_2_ graphene devices. To elucidate the underlying sources of the disorder, we perform finite-element modeling of the electric field. We specifically consider nanohole disorder in the form of nanohole size variations, adjacent nanohole mergers, and nanohole vacancies. Using a resistor network model, we characterize the impact of the disorder on the electrical resistance of graphene. We show that among the various sources, the variation in the nanohole size is the dominant factor in our devices. More specifically, we show that the full-width half maximum (FWHM) of the resistance peak changes by 600% for 5% nanohole size disorder, whereas the FWHM changes by 700% for 5% nanohole size variations and 3% adjacent nanohole mergers. We correlate the theoretically calculated disorder to our experimental results using topography characterization of the SL and find that the increase in disorder of our graphene devices closely matches the theoretical prediction. We also utilize quantum transport simulations coupled with the finite-element modeling to qualitatively confirm the results. This study hence provides a framework to predict and engineer disorder in patterned SLs, specifically correlating variations in the resultant SL patterns to the observed disorder.

## 2. Materials and methods

### 2.1. SL fabrication

The SL is prepared on a 1 cm^2^ Si/SiO_2_ wafer (285 nm dry, thermal oxide; NOVA Wafer) with Cr/Au alignment markers (5 nm/45 nm). The wafers are dehydrated for 20 mins at 180 °C before spin coating a mixture of ZEP520A and anisole (1:1 ratio) at 6000 rpm for 45 s. The samples are then baked on a hot plate for 3 mins at 180 °C. The SL is patterned using EBL (Raith EBPG5200) with a beam current of 500 pA, beam step size of 5 nm, and a dose of 400–435 *µ*C cm^−2^. The pattern is designed to be a square SL (area, *A*^2^ = 40^2^ nm^2^) formed by circular nanoholes (radius, *r =* 12.5 nm) with an etch depth of ∼30 nm. The patterns are developed in n-Amyl acetate at −10 °C for 3 mins followed by rinsing in isopropanol (IPA) for 1 min and drying with N_2_. Dry etching is carried out in a Plasma-Therm Versalock 700 inductively coupled plasma system. The chamber is cleaned for 15 mins under an O_2_ environment at 800 W (ICP power). The SiO_2_ is then etched for 30 s at 5 mTorr in a mixture of CHF_3_ (30 sccm) and CF_4_ (10 sccm) with a chuck power of 50 W and ICP power of 300 W. The depth of the etched regions is ∼30 nm. After etching, the resist is removed in dimethyl sulfoxide heated to ∼80 °C for at least 1 h followed by rinsing in IPA and DI water. To remove any residual resist, the samples are exposed to an O_2_ plasma (Harrick Plasma) for 10 mins at 30 W and 840 mTorr. After plasma cleaning, the samples are further cleaned in Nanostrip heated to 60 °C for 20 mins and thoroughly rinsed with DI water before drying.

### 2.2. Heterostructure assembly

Monolayer graphene (Kish Graphite, CoorsTek Inc.) is mechanically exfoliated and identified via optical contrast. A top graphite is also exfoliated and picked up first followed by the top hBN (15 nm, HQ Graphene), the graphene, and bottom hBN (5 nm). Standard EBL is used to pattern and etch the heterostructure. Another EBL step is performed to define the Cr (10 nm)/Au (100 nm) contacts.

## 3. Results and discussion

### 3.1. Topographic analysis of the nanopatterned SL

**Figure 1.**
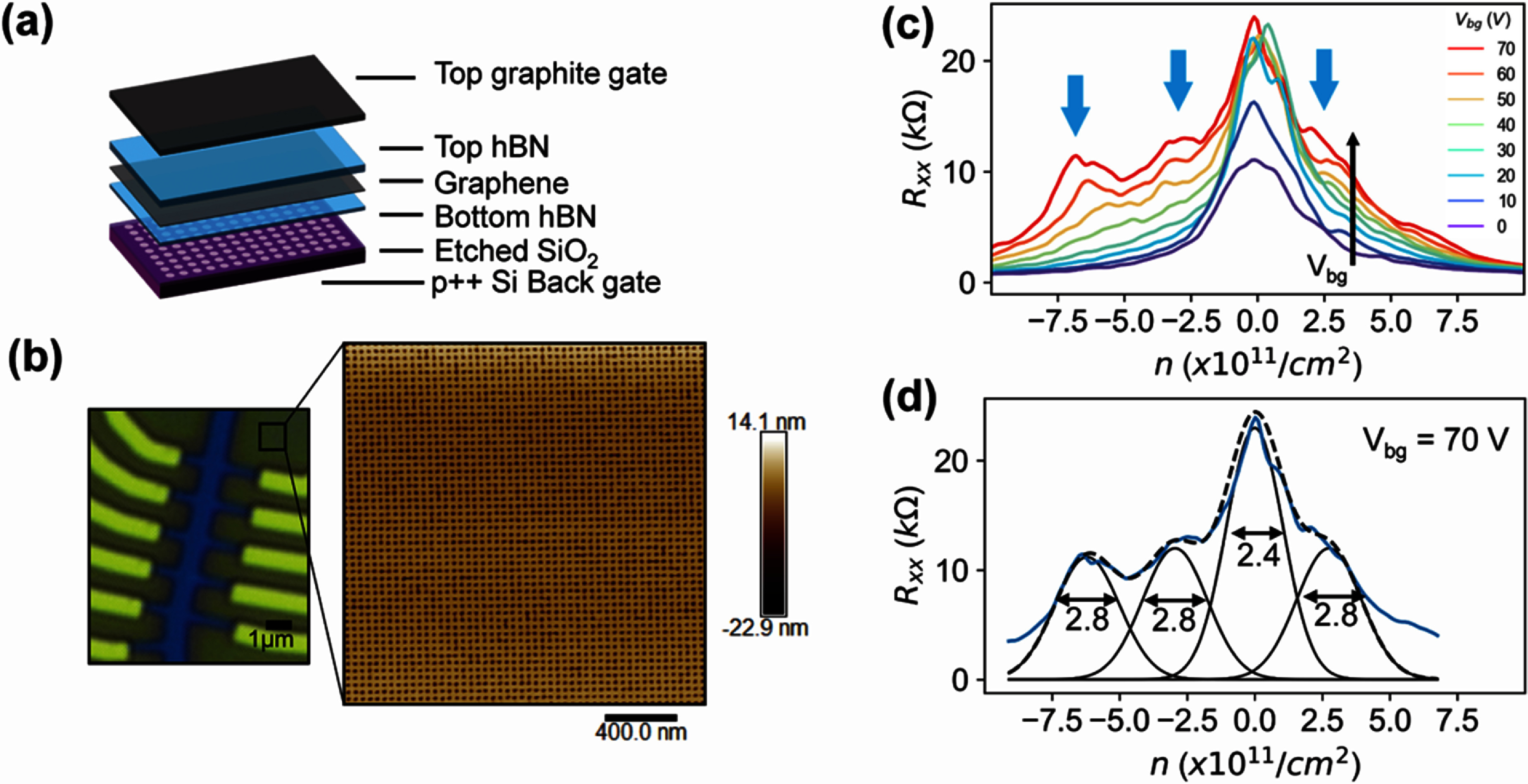
Device structure and electrical transport at zero magnetic field. (a) Schematic of the hBN-encapsulated heterostructure consisting of patterned SiO_2_ as the back gate dielectric and a top graphite gate. (b) Optical image of the fabricated hall bar along with atomic force microscope (AFM) image of the patterned substrate. (c) Longitudinal resistance ${R_{xx}}$ as a function the carrier density $n$ at various back gate voltages (${V_{{\text{bg}}}}$’s). The satellite peaks due to nanopatterned superlattice (SL) are marked by blue arrows. (d) The individual Gaussian peaks fitted to estimate the full width half maximum (FWHM) of each peak. The values of the FWHM are indicated within the panel for each peak.

Figures 1(a) and (b) show the schematic of our nanopatterned SL graphene heterostructure and an optical image of the device, respectively. Figure 1(b) also depicts an atomic force microscope (AFM) image of the nanopatterned SL (high resolution AFM image and other data from this paper is available online [44]). We fabricate a graphite/hBN/graphene/hBN heterostructure on a 285 nm SiO_2_/Si substrate using a standard dry transfer technique. Prior to the heterostructure transfer, we nanopattern the SiO_2_ dielectric into a square-patterned SL, consisting of nanoholes with a diameter of approximately 25 nm and pitch size of approximately 40 nm. We encapsulate graphene with a 5 nm-thick bottom hBN flake which is thin enough to preserve the spatial SL electric field pattern; more information about the device fabrication can be found in the supplementary information (SI). As shown in the AFM image of figure 1(b), we observe a uniform square pattern over a 4 *μ*m^2^ area. We identify two types of defects in the SL from this AFM image. First, using image analysis of the AFM data, we estimate ∼5% variations in the nanoholes size (represented by ${\gamma _r}$); see SI. For this estimation, we assume a normal distribution for the radius of nanoholes with $12.5 \times \frac{{{\gamma _r}}}{{100}}$ nm as the standard deviation and $12.5$ nm as the mean. Second, we observe ∼3% nanohole mergers in the SL when two or more nanoholes have merged. We represent this by ${\gamma _m}$, which is the percentage of the number of mergers per number of nanoholes. We also consider nanohole vacancies where the nanofabrication failed to yield a nanohole. We represent this by ${\gamma _v}$, which is the percentage of the number of vacancies per number of nanoholes. We note, however, that our image analysis does not recognize any vacancies in our patterns. Therefore, we will primarily use ${\gamma _r}$ and ${\gamma _m}$ to compare our experimental results with the theoretical model.

### 3.2. Electrical transport measurements

We perform electrical transport measurements using standard lock-in amplifiers (SR860, Stanford Research Systems) in a Bluefors dilution refrigerator (LD250) with a base temperature of 10 mK. All measurements are performed at 10 mK unless noted otherwise. Figure 1(c) plots the longitudinal resistance (${R_{xx}}$) for various carrier concentrations and back-gate voltages. We calculate the carrier concentration ($n$) using a parallel-plate capacitor model. Specifically, we use the trajectory of the main Dirac point as a function of back- and top-gate voltages to calculate the intrinsic carrier density $n_0^{\prime}$ using a double capacitor model as $n\, = \,{C_{{\text{tg}}}}{V_{{\text{tg}}}}\, + \,{C_{{\text{bg}}}}{V_{{\text{bg}}}}\, + \,n_0^{\prime}$ [25, 36]. By fitting the charge neutrality point ($n$ = 0), we obtain $n_0^{\prime} \approx $ 4.3 $ \times $ 10^11^ cm^−2^; see section S3 of the SI for more details. Figure 1(c) shows the expected main Dirac peak near the charge neutrality point. We further observe mini peaks around the charge neutrality point (blue arrows in figure 1(c)), which only emerge when the SL potential is large (${V_{{\text{bg}}}}\, &gt; \,40{\text{ V}}$). This observation is consistent with previous reports [25], that the satellite peaks are due to the spatially periodic electric field formed by the nanopatterned dielectric. For a square SL, the first satellite peak is predicted to appear at $4{n_0} = \frac{4}{{{A^2}}} = 2.5 \times {10^{11}}$ cm^−2^ where ${A^2}$ is the area of the SL unit cell ($A$ $ \approx $ 40 nm) and ${n_0} = \frac{1}{{{A^2}}}$ [33]. In our device, the first satellite peak occurs at $n = - 2.5 \times {10^{11}}$ cm^−2^ which is consistent with the predicted $ - 4{n_0}$ for a square SL [25]. The second peak, however, appears at $n = - 12{n_0} = - 7.5 \times {10^{11}}$ cm^−2^ which is larger than the expected peak around $\left| n \right| = 6.5{n_0}$ for a square lattice. We note that this disparity has also been observed in previous reports [25], and is attributed to the simplicity of the model which underestimates the lattice strength and hence overestimates the bandgap overlap in the experimental system. Because of this disparity, we perform quantum transport simulations of a scaled graphene device on a square SL (see section 3.5 and the SI). We find theoretically that the two main SL peaks appear at $n = - 4{n_0}$ and $n = - 12{n_0}$, respectively, corroborating our experimental observations. We also perform magneto transport measurements to confirm SL effects as described in the SI. Finally, we note that the observed satellite peaks are weaker on the electron side with only one clear peak. This non-symmetry has previously been observed [25] and is associated to the electron-hole asymmetry of the band structure, resulting in an asymmetry in the resistance vs carrier concentration curves.

To analyse the disorder, we consider the FWHM of the resistance versus carrier density data. We find that the peaks in figure 1(c) are broad (FWHM ∼ $2.4 \times {10^{11}}$ cm^−2^) indicating a high disorder level in our graphene heterostructure. However, this FWHM is comparable to previously reported data for similar devices e.g. FWHM $ \approx $ $2 \times {10^{11}}$ cm^−2^ for 35 nm square SL and FWHM $ \approx $ $3.5 \times {10^{11}}$ cm^−2^ for 40 nm triangular SL [25]. We note that the FWHM is commonly used to analyze disorder in data with a single peak [34]. Since our data contains multiple overlapping peaks, we use a Gaussian distribution for the constituent peaks and fit them to the resistance vs carrier concentration data shown in figure 1(c) for *V*_bg_ = 70 V. The fitted curve closely matches the raw data as seen in figure 1(d). From these constituent peaks, we individually extract the FWHMs as $2.4 \times {10^{11}}$ cm^−2^ and $2.8 \times {10^{11}}$ cm^−2^ for the main peak and the satellite peaks, respectively. This FWHM is an order of magnitude larger than that of graphene devices on non-patterned substrates [34]. This observation serves as a motivation for us to further investigate the sources of disorder in dielectric-patterned SL devices in the later part of the manuscript.

### 3.3. Finite-element modeling

**Figure 2.**
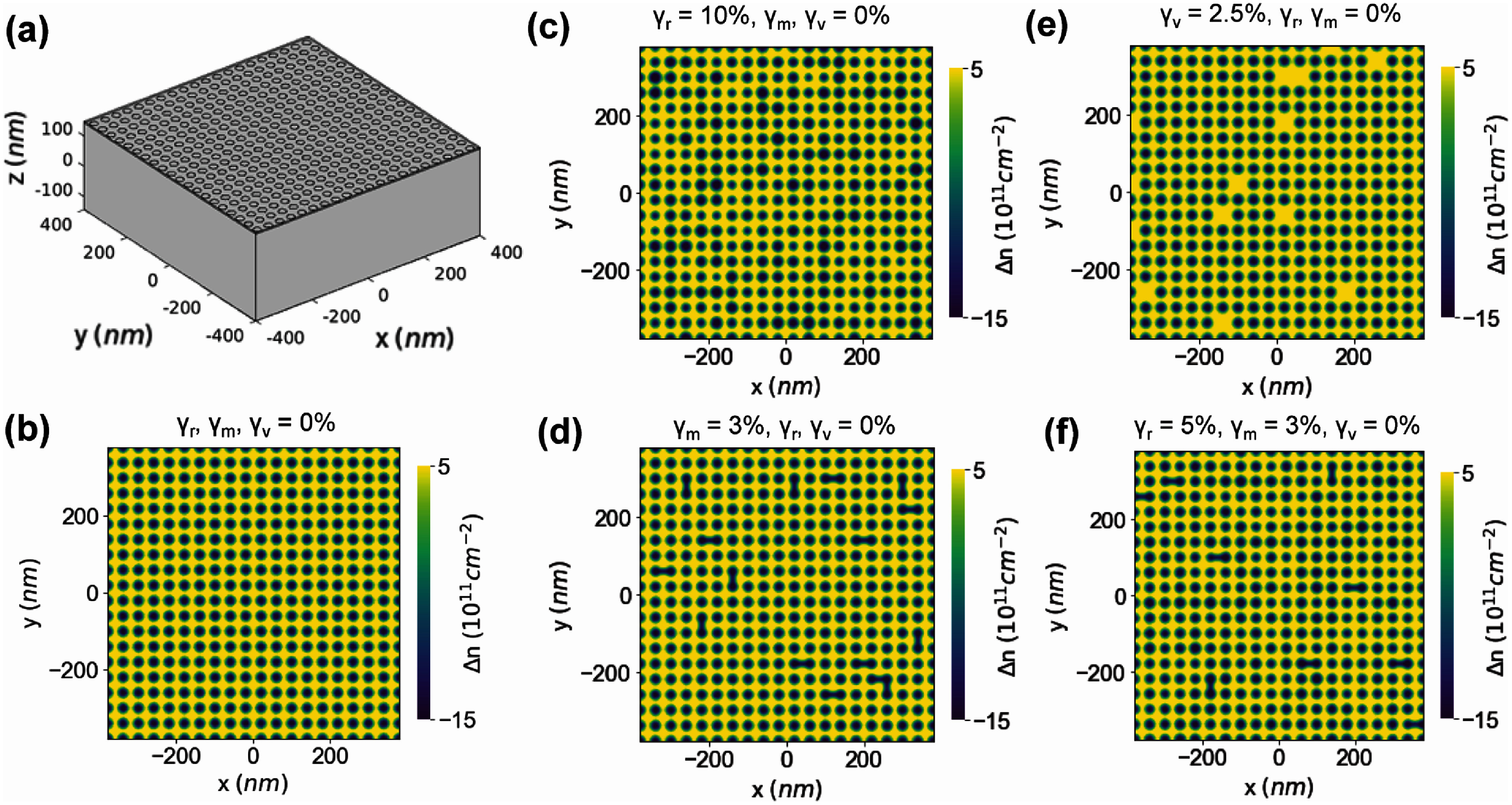
Finite-element modeling. (a) The geometry of a dielectric superlattice with constant radii used in the finite-element analysis. The top hBN layer is removed for clarity. (b) Exemplary maps of differential carrier density $\Delta n$ for a pristine superlattice (no variation in nanohole size ${\gamma _r}$ = 0%, no nanohole vacancy ${\gamma _v}$ = 0%, and no nanohole merger ${\gamma _m}$ = 0%) and a superlattice with randomly varying ${\gamma _r}$ (c), ${\gamma _m}$ (d), and ${\gamma _v}$ (e). (f) The map of ${{\Delta }}n$ corresponding to ${\gamma _r}$ = 5%, ${\gamma _m}$ = 3%, and ${\gamma _v}$ = 0%, corresponding to the topographically estimated variations from our AFM image.

**Figure 3.**
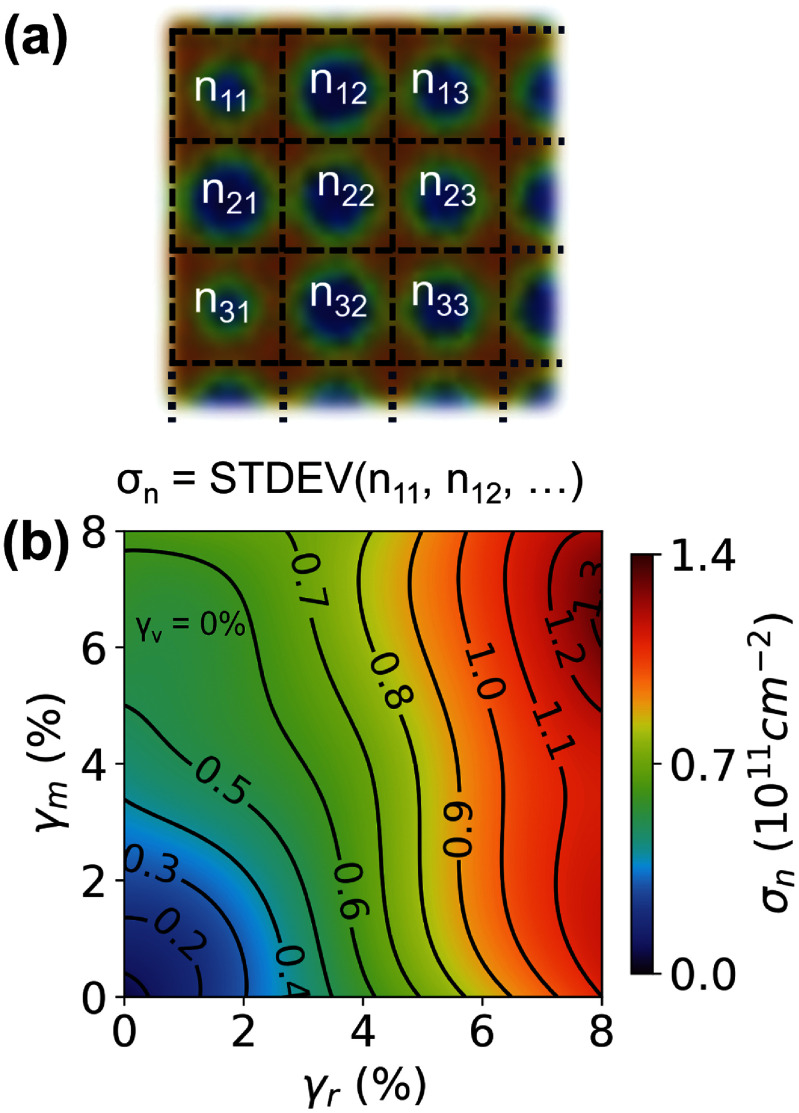
Variations of the carrier concentration in the superlattice. (a) The calculated average carrier density *n_ij_* for each unit-nanohole which is then used to calculate the standard deviation, ${{{\sigma }}_n}$. (b) The standard deviation ${{{\sigma }}_n}$ as a function of ${\gamma _m}$ and ${\gamma _r}$ with ${\gamma _v}$ = 0%.

We perform finite-element modeling using COMSOL Multiphysics version 6.2 and MATLAB version R2023a to better understand how nanolithography limitations in fabricating nanopatterned dielectric SLs affects the electrostatic gating across the sample. We use the Electrostatics module with a physics-based mesh specified to parameter ‘3’. The model comprises a Si back gate (*V*_bg_) as a boundary condition placed at the base of a SiO_2_ dielectric layer (285 nm and dielectric constant *ε* = 3.9). The SL is composed of nanoholes which are treated as ideal vacuum (*ε* = 1) with a depth of 30 nm, a radius (*r*) of 12.5 nm, and a pitch (*a*) of 40 nm. Figure 2(a) shows the simulation geometry, which comprises a 20 $ \times $ 20 array of uniform nanoholes (radius *r* = 12.5 nm, pitch size *a* = 40 nm, depth = 30 nm) etched into a 285 nm thick SiO_2_ volume. We include a 5 nm thick hBN layer (*ε* = 3) on top of the SiO_2_ is and set the boundary condition to ground on top. In this figure, the hBN layer is excluded for clarity. Using a geometric capacitance model, we calculate the carrier concentration ($n$) and differential carrier concentration $\Delta n = n - \langle n\rangle $, where $\langle n\rangle $ is the average carrier concentration across the substrate. We use ${{\Delta }}n$ instead of $n$ in our calculation to highlight the variations in the carrier concentration. We allow the radii of the nanoholes to vary according to a Gaussian distribution centered around the prescribed radius of 12.5 nm and standard deviation of $12.5 \times \frac{{{{{\gamma }}_r}}}{{100}}{\text{ nm}}$, where ${\gamma _r}$ captures nanohole size variations. Moreover, we allow adjacent nanoholes to merge with a percent ${\gamma _m}$ and consider nanohole vacancies by including missing nanoholes with a percent ${\gamma _v}$. Figure 2(b) shows uniform distribution of $\Delta n$ for ${\gamma _r}$, ${\gamma _v}$, and ${\gamma _m}$ = 0%, i.e. a pristine SL. On the other hand, figures 2(c)–(f) show the distribution of ${{\Delta }}n$ for different combinations of ${\gamma _r}$, ${\gamma _v}$, and ${\gamma _m}$. Such variabilities in ${\gamma _r}$, ${\gamma _v}$, and ${\gamma _m}$ could arise in practice from minor instabilities and fluctuations during nanolithography. In this case, spatial inhomogeneity of the displacement field caused by non-zero ${\gamma _r}$, ${\gamma _v}$, and ${\gamma _m}$ leads to nonuniformity in the induced carrier concentration of graphene. To better quantify ${\gamma _r}$, ${\gamma _v}$, and ${\gamma _m}$ in our simulated SL, we calculate the average carrier density *n_ij_* for each of the unit-nanohole, i.e. a 40 nm by 40 nm square region as shown with dashed squares in figure 3(a). This formulation allows us to look at the variation of the carrier density in each unit cell due to nanohole imperfections. Since our substrate has no nanohole vacancies, we focus on ${\gamma _r}$ and ${\gamma _m}$. We determine ${{{\sigma }}_n}$ which is the standard deviation of *n_ij_* for various ${\gamma _r}$ and ${\gamma _m}$ (${\gamma _v}$ = 0%) and plot it in figure 3(b) using contour lines for visual clarity. We observe that ${{{\sigma }}_n}$ is close to zero for pristine SL (${\gamma _r}$, ${\gamma _v}$, ${\gamma _m}$ = 0%) as expected. As ${\gamma _m}$ increases from 0% to 8%, we observe an increase of approximately $0.6 \times {10^{11}}$ cm^−2^ in ${{{\sigma }}_n}$, whereas, we observe a larger increase of approximately $1.1 \times {10^{11}}$ cm^−2^ when ${\gamma _r}$ increases from 0% to 8%. At low ${\gamma _m}$ < 3%, the effect of ${\gamma _m}$ and ${\gamma _r}$ is similar when compared independently. However, at higher levels (${\gamma _m} &gt; 3\% $), ${\gamma _r}$ is dominant. For ${\gamma _r}$ = 5% and ${\gamma _m}$ = 3% estimated from our AFM data, ${{{\sigma }}_n}$ is roughly $0.8 \times {10^{11}}$ cm^−2^. We will now discuss how this standard deviation (${\sigma _n}$) is translated to an estimate for the FWHM.

### 3.4. Resistor network simulation

**Figure 4.**
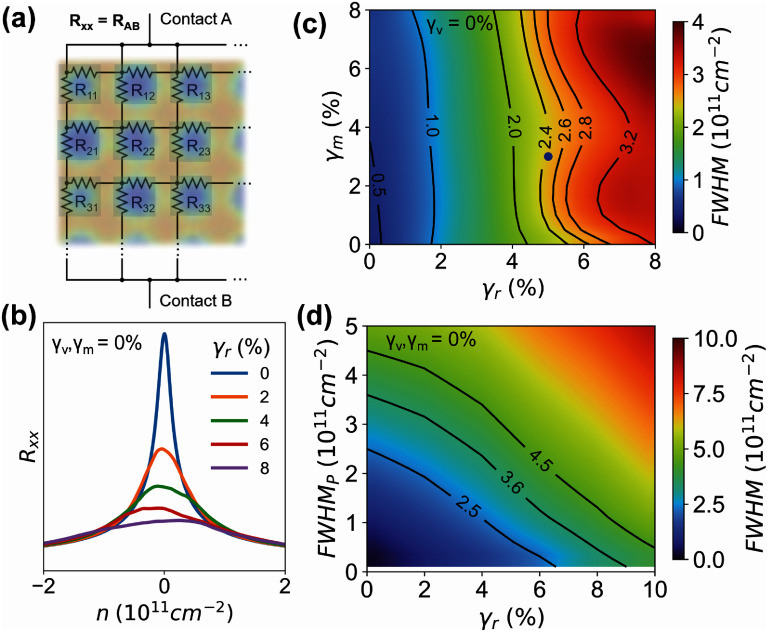
Resistor network model to estimate disorder. (a) Schematic of the resistor network used to simulate disorder. (b) Calculated ${R_{xx}}$ versus $n$ for varying ${\gamma _r}$. (c) FWHM of the primary Dirac peak as a function of ${\gamma _r}$ and ${\gamma _m}$. Blue dot represents the estimated disorder for our experimental superlattice device. (d) FWHM of the primary Dirac peak versus ${\gamma _r}$ and the FWHM_P_ of pristine graphene.

To include the effects of nanohole disorder in the resistance, we model graphene using a resistor network, which has been successfully used to model charge puddles in graphene [45, 46]. We simulate a square network of resistors as shown in figure 4(a), wherein each node is connected to the right and bottom nodes via two similar resistors (*R_ij_*). We determine *R_ij_* for each unit cell using the function *R*(*n = n_ij_ + n*_tg_). The resistance *R* is analytically modeled based on a response of a miniscule graphene device with FWHM of ∼$0.25 \times {10^{11}}$ cm^−2^ (see section S7 for analytical expression of *R* and a plot of *R* vs *n* behaviour). In our system, the intrinsic disorder may be present due to the charge puddles at the interface of the encapsulated hBN-graphene-hBN heterostructure and the silicon dioxide substrate. The value of the FWHM depends on the substrate and can be changed based on the engineered device characteristics. For example, the FWHM may be higher when graphene is directly placed on SiO_2_ substrate [35, 47], or lower when graphite gates are used which screen charges at the SiO_2_ interface [48]. To calculate the value of *R*, we plug in *n* = *n_ij_* + *n*_tg_. *n_ij_* is the average unit cell carrier density defined earlier and *n*_tg_ is the carrier density induced by the top gate. This *n_ij_* depends on the back-gate voltage, and more importantly captures the variations in the SL patterns (see figure 3). We calculate the local carrier concentration *n_ij_* based on the finite-element simulation. *n*_tg_ is the global carrier concentration induced by the top gate, which we assume is uniform throughout the substrate at a fixed top gate voltage. The local carrier concentration, however, *n_ij_* is in fact not uniform in a non-uniform pattern and can vary across the substrate based on nanopattern variations. We note that varying ${\gamma _r}$, ${\gamma _v}$, and ${\gamma _m}$ results in changes in *n_ij_* which will be captured by the function *R* (*n = n_ij_ + n*_tg_). We calculate the resistance using the network of figure 4(a) and plot *R_xx_* versus *n* in figure 4(b). In order to calculate the *R_xx_* of the macroscopic device, *R_ij_* of each microscopic graphene device with varying *n_ij_* is calculated and simulated using a resistor network. The more variation present in the *n_ij_* more the *R_xx_* peak broadens due to misalignment of the charge neutrality points of the microscopic graphene devices. We observe that for ${\gamma _r}$ = 0%, full width half maximum is FWHM_P_ = $0.35 \times {10^{11}}$ cm^−2^ which is slightly higher than the expected value of $0.25 \times {10^{11}}$ cm^−2^. We attribute this discrepancy to the numerical inaccuracy due to digitization of large carrier concentration numbers in the finite-element simulation.

### 3.5. Quantum transport simulation and outlook

We observe that in figure 4(b), with an increase in ${\gamma _r}$, the main Dirac peak broadens; this is because each SL unit cell has a slightly different carrier concentration (${n_{ij}}$). As a result, the calculated FWHM increases with the increasing ${\gamma _r}$. Figure 4(c) plots the colormap of FWHM as a function of ${\gamma _r}$ and ${\gamma _m}$. Solid black lines with labels correspond to contours with fixed FWHM with varying ${\gamma _r}$ and ${\gamma _m}$. We observe that the constant FWHM contours are generally vertical. This indicates that ${\gamma _r}$ has a greater influence on the FWHM. We further observe that the FWHM increases by as high as 1000% for ${\gamma _r}$ and ${\gamma _m}$ around 8% when compared to FWHM_P_. For our experimental conditions of ${\gamma _r}$ = 5% and ${\gamma _m}$ = 3%, our model predicts the FWHM of ∼$2.4 \times {10^{11}}$ cm^−2^ which is close to the experimentally observed FWHM (blue dot in figure 4(c). In our device, ${\gamma _r}$ = 5% alone corresponds to ∼600% increase in FWHM compared to FWHM_P_. When ${\gamma _m}$ is also increased from 0% to 3%, the FWHM increases by ∼700%. This FWHM matches the one we estimate from our experimental results in figure 1(c). To further confirm the results of our theoretical modeling, we perform quantum transport simulations where we use the output of the finite-element model as the SL potential input. In this case, we simulate a 0.5 *µ*m^2^ square area scaled graphene (scaling parameter = 4) lattice using a tight-binding model [49]. We perform all numerical calculations using a python-based Kwant code [50]. We consider two leads which are attached to the opposite sides of the square lattice. We calculate the SL potential from the carrier concentration obtained via the finite-element simulation. The SL potential nominally varies with a peak–peak value of 50 meV which is comparable to experiments for back-gated graphene devices with patterned dielectric [25, 31, 51]. To induce intrinsic disorder, we delete 0.01% of carbon atoms to form vacancies. We utilize a Savitzky–Golay filter (sampling distance of 0.1 meV, 35 window size, 2nd order) to smoothen the noisy data around the Dirac peak and to better estimate the relative FWHM. This noise is due to numerical variation of the SL potential from the finite-element simulation. Figure S5 of the SI shows the schematic of the quantum simulation and plots the calculated transmission as a function of carrier density and energy. By varying the disorder, especially ${\gamma _r}$ from 0% to 4%, we observe an increase in FWHM as the disorder increases; see figure S5(c) in the SI. Overall, we calculate a 3× increase in FWHM from FWHM_P_ at ${\gamma _r}$ = 4%. This is comparable to our predicted value when FWHM_P_ is around ${10^{10{\text{ }}}}$ cm^−2^.

Figure 4(d) plots the colormap of the FWHM as a function ${\gamma _r}$ and FWHM_P_. We observe that the FWHM increases with increasing ${\gamma _r}$. Assuming the maximum tolerance of the FWHM to be around $4{n_0}$, we can estimate the maximum allowed FWHM_P_ for each ${\gamma _r}$. For example, for a square SL of 40 nm wavelength, i.e. $4{n_0} = 2.5 \times {10^{11}}$ cm^−2^, the maximum allowed ${\gamma _r}$ is around 6.5% for a high-quality graphene device with FWHM_P_ $\sim {\text{ }}{10^{10}}$ cm^−2^. In fact, figure 4(d) can be used as a guide to estimate the expected disorder in the system by assuming a reasonable FWHM_P_ and measuring the ${\gamma _r}$ prior to heterostructure fabrication. This will allow us to predict if the SL pattern will yield a device with low enough disorder. Therefore, assuming a state-of-the-art EBL limit of 30 nm in feature dimensions [9] and FWHM_P_ = 10^10^ cm^−2^, we estimate a maximum allowed ${\gamma _r}$ = 11% to achieve the FWHM of $4.5 \times {10^{11}}$ cm^−2^ $ \unicode{x2A7D} 4{n_0}$. Our model, although described for a square SL of 40 nm wavelength, can easily be adapted to other SL geometries; see for example the results for a triangular lattice in section S8 of the SI. In contrast to square SL, we observe that ${{{\sigma }}_n}$ depends equally on both ${\gamma _r}$ and ${\gamma _m}$. This is because in a triangular lattice, each nanohole has six neighboring nanoholes and a higher chance of nanohole fusion. Moreover, due to the smaller relative unit cell compared to a square lattice, each fusion leads to a higher susceptibility in the variation of the carrier concentration. Interestingly, we observe that triangular SL has higher resistance towards nanohole size variations as compared to the square lattice. This observation together with the fact that for the same wavelength, $4{n_0}$ in triangular SLs is much higher than square SLs make triangular SLs more robust against disorder. Although not a replacement for quantum simulations, we speculate our model is a simple and versatile tool to understand and evaluate disorder in nanopatterned SL devices.

## 4. Conclusion

In conclusion, we successfully demonstrated the use of a patterned dielectric SL to fabricate a device with *in-situ* tunable SL effects. We performed longitudinal resistance and Hall measurements to confirm the presence of the SL effects. We observed that the SL device had a higher disorder compared to unpatterned graphene devices. We investigated this disorder through a combination of modeling and experimental analysis. Specifically, we investigated three kinds of disorder: variations in the size of nanoholes, adjacent nanohole mergers, and nanohole vacancies. Furthermore, we modeled graphene using a resistor network to translate the variations in the simulated electrostatics to disorder in transport characteristics. We found that for square SLs, the disorder primarily originates from variations in the SL pattern formed during lithography. We further confirmed this finding using a quantum transport simulation model. The developed disorder model could offer new insights into ways to improve the SL quality in nanopatterned devices. Furthermore, beyond graphene, the developed model could be generalized to study other 2D materials and device structures for a variety of electronic applications. Our combined experimental and theoretical results could help determine the accepted disorder level prior to complex nanofabrication of 2D heterostructures with nanopatterned dielectric layers or gate electrodes.

## Data Availability

The data that support the findings of this study are openly available at the following URL/DOI: https://doi.org/10.5281/zenodo.13221419.
